# An Anatomical Study on Dominant Vascular Pedicle of Tibialis Anterior Muscle and its Implication on Tibialis Anterior Muscle Flap

**DOI:** 10.1055/s-0045-1802631

**Published:** 2025-02-10

**Authors:** Venkatesh M.S, Shwetha B.M, Manjunath K.N, Ashwini Shivaprasad, Veena Vidyashankar, Anupama K.

**Affiliations:** 1 Department of Plastic Surgery, Ramaiah Medical College and Hospital Pvt. Ltd., Bangalore, Karnataka, India; 2 Anatomy Department, Ramaiah Medical College and Hospital Pvt. Ltd., Bangalore, Karnataka, India

**Keywords:** tibialis anterior, dominant vascular pedicle, TAMF, function sparing

## Abstract

**Background:**

The tibialis anterior muscle flap (TAMF) is a reliable option to provide coverage for the middle third of the leg. Identification of multiple segmental vessels for the TAMF often proves to be a tedious procedure. A single dominant vascular pedicle, if identified, can be consistently used for harvesting the TAMF. There are no anatomical studies in the literature that propose to identify the main vascular pedicle of the tibialis anterior muscle, which can be consistently used for transfer.

**Materials and Methods:**

Forty lower limbs of 20 cadavers were used for the study. Microdissection of the limbs was done to identify the tibialis anterior muscle and the vessel along their entire length. The number of vascular pedicles and the location of each pedicle from the knee joint line and tibial tuberosity were noted.

**Results:**

There were a mean of 7.45 (minimum: 5; maximum: 9) segmental perforators from the anterior tibial artery. The average diameter of the dominant perforator was 1.10 ± 0.12 mm. The mean distance of the dominant pedicle from the knee joint line and the tibial tuberosity was 12.15 ± 0.98 and 7.7 ± 1.8 cm, respectively.

**Conclusion:**

The dominant vascular pedicle of the tibialis anterior muscle is consistently found at an average distance of 12 cm from the knee joint line and 7.5 cm from the tibial tuberosity. The dominant pedicle could perfuse about 70% of the muscle bulk. A partial TAMF can be devised based on this dominant pedicle for middle one-third leg defects.

## Introduction

Exposed bone in the lower extremity, especially the leg, is a challenging clinical scenario faced by plastic and reconstructive surgeons in everyday practice. The primary objective in such circumstances is to restore and maintain optimum extremity function. 1 For reconstructive purposes, the lower limb can be divided into five zones: thigh, knee, middle leg, lower leg, and foot and ankle. Traditional reconstructive armamentarium suggests the use of local muscle flap for the upper two-thirds of the leg and free tissue transfers for the distal leg and foot. 2 The best local flap is one that is technically simple, done in a single-stage surgical procedure, and has minimal donor-site morbidity, giving a good functional and aesthetic outcome. 3 When encountered with a linear defect in the middle or distal third of the leg, the bulky posterior compartment muscles may be inappropriate, which prompts the surgeons to use other minor leg muscles for coverage. 4 The tibialis anterior (TA) muscle is a muscle that lies superficially in the anterior compartment of the leg, just lateral to the tibia and medial to the extensor hallucis longus (EHL) and extensor digitorum longus (EDL) muscles. 5 The TA muscle originates from the lateral condyle of the tibia, upper lateral surface of the adjacent tibia, interosseous membrane, and crural fascia, and it inserts into the medial cuneiform and base of the first metatarsal of the foot. 6 The TA muscle is a strong dorsiflexor and invertor of the foot that makes it an indispensable muscle. 7 However, there are numerous function preservation techniques like superior-based rotation flap, anterior turnover flap, posterior advancement flap, and sagittal split method, which have all been successfully described in the literature for harvesting the tibialis anterior muscle flap (TAMF). 4 8 9 10 11 The TAMF can be a reliable option to provide coverage for the middle and distal thirds of the leg if function-sparing techniques are used for its harvest. The TA muscle is classified as type IV muscle according to the Mathes and Nahai classification. 12 Identification of these multiple segmental vessels for the TAMF often proves to be a tedious procedure. A single dominant vascular pedicle, if identified, can be consistently used for harvesting the TAMF. There are no anatomical studies in the literature that propose to identify the main vascular pedicle of the TA muscle that can be consistently used for transfer. The present anatomical study aims to identify the most dominant vascular pedicle of the TA muscle and its location. The same will be utilized to devise a partial TAMF.

## Materials and Methods

Twenty cadavers with 40 lower limbs from the Advanced Learning Centre (ALC) of our institute were used for the study. The cadavers were preserved by the modified Thiel embalming, which retains the flexible properties of the human bodies. The silicone dye injection also helps enhance visibility, thus not losing out on important vascular structures. Anthropometric data of the cadavers like age, sex, and height of the cadaver were noted. In addition, other measurements like lower limb length, leg length, and leg circumference were documented.

## Dye Study

Liquid silicone dye was injected into the anterior tibial artery (ATA) after ligation of the popliteal vessel (right and left selected randomly). The injected cadavers were positioned in the upright position for 24 hours to allow solidification of the dye. After 24 hours, microdissection under 4x loupe magnification of the anterior compartment of the injected limbs was done to identify the TA muscle and the vessel (ATA) along their entire length. The perforator with the maximum visible diameter was selected. The location of the same was measured from the bony landmarks, the tibial tuberosity and the knee joint line proximally and the medial malleoli distally. The diameter of the perforator was measured at the origin using digital calipers up to 2 dB in millimeters. Descriptive statistics of the dominant vascular pedicle like the diameter and its location were summarized in terms of the mean and standard deviation.

## Perfusion Study

The noninjected sides of the same 20 cadavers were utilized. The TA muscle was harvested along with the ATA. As per the opposite limb dye study, the largest diameter perforator was identified and the ATA vessel was ligated distal to the perforator. Urografin dye was slowly injected into the ATA proximally using a 2-mL syringe. The perfusion of the muscle was monitored using fluoroscopy. The fluoroscopy images are obtained and approximate area of perfusion was noted as the percentage of the muscle perfused.

## Results

Six male and 14 female cadavers with an average age of 81.9 years were dissected. The average cadaver length and the lower limb length were 157.4 and 82.9 cm, respectively ( Table 1 ).

**Table 1:** Anthropometric measurements of the study

Anthropometric parameters	Value
Male	6
Female	14
Age (y)	81.9 ± 5.1
Right and left legs ( *N* )	20 each
Cadaver length (cm)	157.4 ± 8
Lower limb length (cm)	82.9 ± 4.5
Leg length (cm)	37.8 ± 2.1
Leg circumference (cm)	23.9 ± 5

The total number of segmental perforators varied from five to nine (maximum number of cadavers had 7 perforators). The largest perforator was found in the proximal third of the leg. The location was 12.15 ± 0.98 cm from the knee joint line and 7.7 ± 1.8 cm from the tibial tuberosity. The average diameter of the dominant pedicle was 1.10 ± 0.12 mm. The size of the limb had no correlation with the diameter of the perforator. However, a positive correlation was found between the limb length and the diameter of the dominant perforator (as the limb length increased, perforator diameter also increased). On perfusion study, 70% of the muscle was perfused. There was no correlation of the same with the girth of the muscle ( Table 2 ).

**Table 2:** Anatomical parameters of the study

Anatomical and perfusion parameters	Value
No. of segmental vessels	7.45 ± 1.1 (minimum: 5; maximum: 9)
Diameter of the dominant pedicle (mm)	1.10 ± 0.12
Location of the dominant pedicle from the knee joint line	12.15 ± 0.98
Location of the dominant pedicle from the tibial tuberosity	7.7 ± 1.8
Muscle perfusion on fluoroscopy (%)	70 ± 5

## Discussion

The TA muscle is a circumpennate muscle situated superficially in the anterior compartment of the leg between the lateral aspect of the tibia and the EHL and EDL muscles. Hirshowitz et al described that blood supply to the muscle originates from the ATA through 8 to 12 segmental vessels. 13 Hence, Mathes and Nahai 12 described the TA muscle as a type 4 muscle. However, in our cadaver study, we found five to nine vessels supplying the muscle. This can be attributed to the length of the limb in our cadavers, which in comparison to the western population is less. A cadaveric study in the Indian population found an average of six or more pedicles, which is similar to our study findings. 14 Even in Indian subcontinent, there can be variation in the length of the cadavers attributable to the racial and ethnic group diversity. Since the study was conducted in single center with cadavers from the same geographic area, such variations could not be assessed. The length of the limb in our study had a positive correlation with the diameter of the ATA at the origin. A similar correlation has been described by Çandır et al in their cadaveric study. 15

**Fig. 1:**
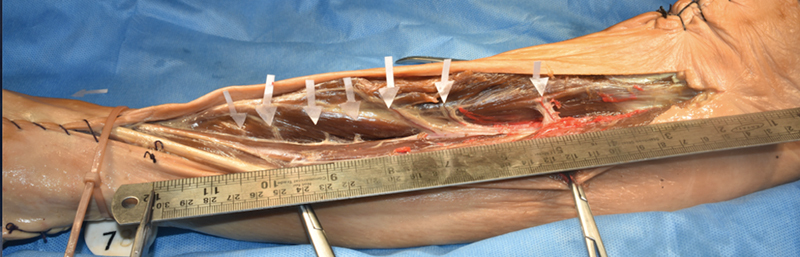
A dissected cadaveric specimen showing the tibialis anterior (TA) muscle with the seven perforators ( *white arrows* ) arising from the anterior tibial artery (ATA). Measurement of the distance of the perforators from the tibial tuberosity and the knee joint line done with a ruler.

It is logical to think that the larger the artery, the greater the supply. Scientific evidence also implies the same. Shatari et al performed a cadaveric study on the gracilis muscle and they found the mean maximum diameter of the major vascular pedicle as 1.08 mm, which is comparable to our study findings. 16 A similar study of the sartorius muscle also showed that the muscle can be harvested on a single pedicle. 17 In our study, the largest diameter visible on loupe magnification was measured using digital calipers after dye injection. In the present study, the dominant vascular pedicle measured about 1.1 ± 0.12 mm and was found consistently at a distance of about 12.15 ± 0.98 cm from the knee joint line and at a distance of about 7.7 ± 1.8 cm from the tibial tuberosity ( Fig. 1 ).

**Fig. 2:**
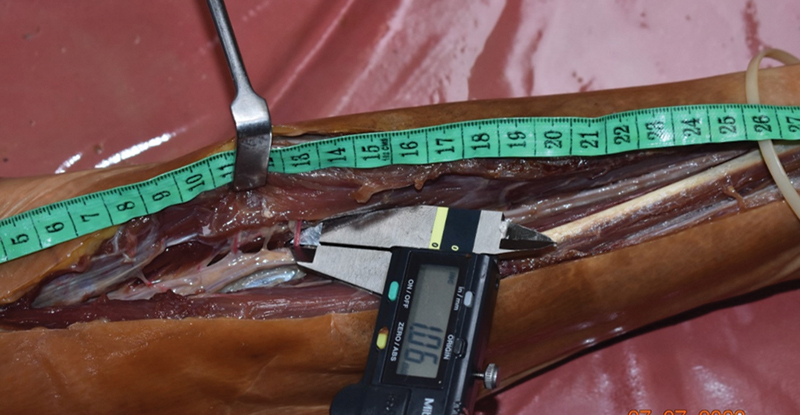
A digital caliper used for measuring the diameter of the vascular pedicle.

The diameter of the largest pedicle in our study was 1.1 ± 0.12 mm. A literature search conducted in both the PubMed and Cochrane databases for the search words “pedicles of tibialis anterior muscle” did not yield any results. Hence, the measurements could not be compared. Age of the cadavers (average 81.9 years in our study) and hence the possibility of arterial disease was a confounding factor. However, by selecting the cadavers that had no resistance to the flow of liquid silicone dye (the viscosity of the dye is more than the blood), we could minimize the chances of arterial diseases in the study cadavers. The surface marking of the dominant pedicle was found at 12.15 ± 0.98 cm from the knee joint line, which can guide the surgeons while harvesting the TA flap. The diameter of the vascular pedicle, which was the largest on the loupe magnification, was measured. The accuracy of measurement of the diameter of the vascular pedicle was ensured by the use of a digital calipers ( Fig. 2 ).

The caliber of the pedicle was not affected by the diameter of the ATA or the side of the limb. However, the length of the limb had a positive correlation with the diameter of the perforator. This indicates that as the limb length increases, there is slight increase in the diameter of the perforator.

**Fig. 3:**
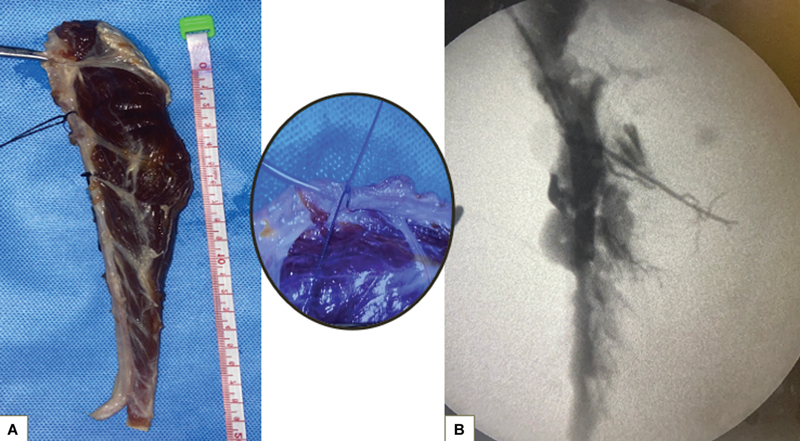
( **A** ) Specimen of the tibialis anterior (TA) muscle with the anterior tibial artery (ATA). An infant feeding tube is inserted into the ATA and ligated distal to the dominant pedicle. ( **B** ) Fluoroscopic image showing significant muscle tissue perfused by the dominant vessel.

**Fig. 4:**
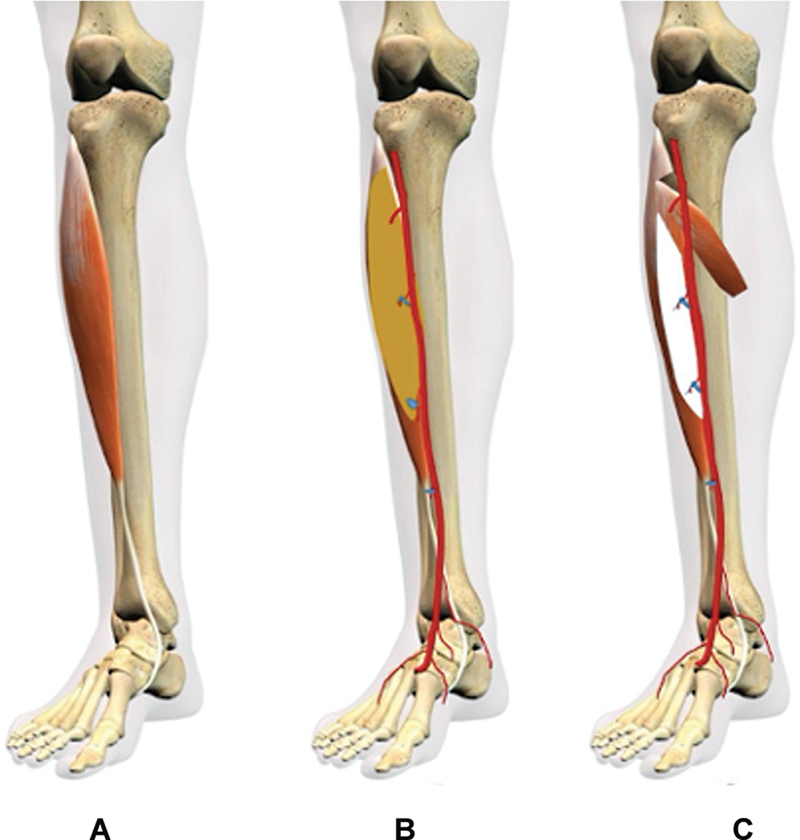
( **A** ) Blood supply of the tibialis anterior muscle. ( **B** ) Amount of muscle perfused on fluoroscopy. ( **C** ) Possibility of muscle flap based on the dominant pedicle.

Robbins et al 18 first described raising a fasciomuscular flap of the TA muscle for covering tibial bone defects. Mathes and Nahai 8 further improvised the concept and utilized a partial muscle harvest for function preservation. Subsequently multiple authors 4 6 9 10 11 13 19 20 have proposed and utilized various function preservation techniques for harvesting the TA muscle. However, all the techniques described are based on harvesting multiple pedicles arising from the anterior tibial vessels. This limits the arc of rotation of the flap. In the literature search also, the vascular supply of the TA muscle has not been studied in the recent years. Since in our cadaveric study we could identify the largest diameter pedicle, we endeavored to further identify the amount of muscle perfused by the single pedicle using radiocontrast perfusion study. We found that 70% of the muscle was perfused by that single largest perforator ( Fig. 3 ). These findings can be a guide to a clinical application. As per the Mathes and Nahai classification, the blood supply is segmental; if the above findings are proved by large number of multicenter study, then the partial TA muscle can be harvested as flap ( Fig. 4 ). Verma in their cadaveric study on the TA muscle noted that the average muscle belly length was 29 cm with a width of 2.65 cm. 14 Considering this dimension, a partial muscle belly harvested based on the single pedicle can be used in cases of small defects of the middle one-third of the leg. However, the exact size can be determined only after successful clinical study, which we intend to do in our next stage. Our study findings provide preliminary supportive evidence for the fact that partial TAMF can be harvested based on a single dominant vessel, but will need further clinical studies.

## Conclusion

The TA muscle is a strong dorsiflexor and an indispensable muscle. However, the TA muscle has good bulk and serves as an alternate to the gastrocnemius and sartorius muscles for small defects of the middle third of the leg. The dominant vascular pedicle of the TA muscle is consistently found at an average distance of 12 cm from the knee joint line and 7.5 cm from the tibial tuberosity. The dominant vessel in our study could vascularize about 70% of the TA muscle bulk. A partial TA muscle flap can be devised based on this dominant pedicle for the middle one-third leg defects if clinically proven.
